# Ensuring Access to HIV Prevention Services in South African HIV Vaccine Trials: Correspondence Between Guidelines and Practices

**DOI:** 10.1093/phe/phu010

**Published:** 2014-06-19

**Authors:** Zaynab Essack

**Affiliations:** HIV AIDS Vaccines Ethics Group (HAVEG), School of Applied Human Sciences, College of Humanities, University of KwaZulu-Natal, Pietermaritzburg, South Africa

## Abstract

Researchers and sponsors are required to assist HIV prevention trial participants to remain HIV-uninfected by ensuring access to prevention services. Ethics guidelines require that these HIV risk-reduction services be state of the art. This and related ethics recommendations have been intensely debated. This descriptive study aimed to identify actual HIV prevention practices for two HIV vaccine trials at five South African sites, to explore whether actual practices meet guideline recommendations and to discuss implications for practices and ethics guidelines. Practices were examined through a review of site documents and interviews with site staff and network representatives, as well as community advisory board and research ethics committee representatives. A thematic analysis of HIV prevention practices, perspectives and perceived challenges was undertaken. Findings indicated that there was a high degree of correspondence between actual practices in South African HIV vaccine trials and guideline recommendations. Key challenges for implementing prevention services were identified as partnerships, provider-promotion of services and participant uptake of services. Practices deviated most from guidelines with regard to the description of prevention plans in informed consent forms. Recommendations are made for both practices and ethics guidelines.

## Introduction

South Africa is home to the highest number of people living with HIV/AIDS. Therefore, there is a public health imperative to develop new and effective HIV prevention methods. Accordingly, South Africa has become a hub for HIV prevention research, including trials of HIV vaccines, microbicides, pre-exposure prophylaxis (PrEP) and male circumcision.

All HIV prevention trials enrol HIV-negative participants. In late-phase trials, participants are at high risk for HIV infection. To reduce risk, participants are provided with access to HIV prevention interventions, recently termed the standard of prevention. The standard of prevention is controversial (Macklin, 2008), with several topics of debate, including norms in ethics guidelines (Essack *et al.*, 2010; Philpott *et al.*, 2011).

There has been debate about what services should be included in the HIV prevention package (Macklin, 2008). While there is broad agreement that participants should receive access to certain prevention interventions (such as condoms, counselling and sexually transmitted infection (STI) treatment), there has been some disagreement about obligations to ensure access to interventions such as male circumcision (Lie *et al.*, 2006), post-exposure prophylaxis (PEP) (UNAIDS, 2000) and PrEP (McEnery, 2012). An additional complexity relates to who should assume the burden for ensuring access to prevention interventions, with some arguing for shared responsibility among sponsors, researchers and host governments (Macklin, 2008; UNAIDS, 2012^1^).

Current ethics guidelines assert that participants should receive ‘optimal’ (SAMRC, 2003) or ‘state-of-the-art’ (UNAIDS-AVAC, 2011; UNAIDS, 2012) HIV risk-reduction interventions. However, some contend that the ‘state-of-the-art’ standard may be too aspirational and not practically feasible (HPTN, 2009; Macklin, 2009) especially in resource-constrained contexts with limited access to high-quality prevention modalities (Macklin, 2010). Ethics guidelines also recommend inter-stakeholder collaboration, and numerous engagement activities, to ensure access to the highest standard of prevention (SAMRC, 2003; UNAIDS-AVAC, 2011; UNAIDS, 2012). Guidelines make a range of recommendations about the standard of prevention, including what should be declared in protocols and informed consent forms; that prevention interventions should be monitored; and about how standard of prevention decisions should be made. These recommendations have been argued to set a very high standard (Essack *et al.*, 2010).

There is little existing data on whether ethics recommendations are being implemented in HIV vaccine trials (HVTs), or on the complexities faced by trial implementers. Commentators have called for an exploration of ‘the prevention services offered to HVT participants’ (Essack *et al.*, 2010, p. 46) and an assessment of the extent to which actual practice (what *is* happening) corresponds with ethics guidance (what *ought* to be happening according to norms) (Macklin, 2010). Such data could respond to the criticism that ethics guidelines represent ideals that cannot be realistically achieved in practice (Macklin, 2010). Some previous research has been conducted on standards of prevention in related HIV prevention trials (Heise *et al.*, 2008; Ngongo *et al.*, 2012). The prior exploration on HVTs (Ngongo *et al.*, 2012), however, aimed primarily to document practices and not to compare practices with ethics recommendations.

## Aims and Methods

This study was one component of a larger study exploring HIV prevention and ancillary care in South African HVTs. This component aimed to explore practices for ensuring preventive methods in two HVTs conducted at five South African sites; establish whether reported practices correspond with related ethics recommendations; identify perceived challenges with service provision; and assess whether ethics guidance addresses identified concerns. It also explored practices related to making decisions about the standard of prevention, but these data are not reported here. Ethics guidelines were selected that governed South African HVTs specifically (SAMRC, 2003) and directly applicable international guidance (UNAIDS-AVAC, 2011; UNAIDS, 2012).

At the time of data collection, five sites were conducting two preventive HVTs. The phase I trial, conducted at two sites, investigated the safety of a vaccine in HIV-negative participants at low risk for HIV. The phase IIB trial, conducted at five sites, investigated the safety and efficacy of a vaccine in HIV-negative participants at high risk for HIV.

Given the exploratory nature of this study, a qualitative approach was adopted. HIV prevention practices were explored by (i) reviewing documents (e.g., protocols, informed consent forms) and (ii) conducting semi-structured interviews with key stakeholders involved in HVTs. Ethics approval was obtained from the research ethics committee (REC) at the researcher’s home institution (University of KwaZulu-Natal’s Biomedical Research Ethics Committee approval number REF BE 241/09) and from three RECs with jurisdiction over the HVT sites. Permission was also provided by the sponsor and principal investigators to access protocols and related trial documents.

Data on prevention practices and perceived challenges came from prevention-specific interviews with site staff [*n* = 13] and network representatives [*n* = 2] as well as from combined prevention and care interviews with community advisory board (CAB) representatives [*n* = 6], REC members [*n* = 8] and site staff [*n* = 1]. Additional relevant data came from care-specific interviews [*n* = 14] where prevention-related references were made. An analysis of care practices and perspectives at SA HVT sites is published elsewhere (Slack, 2014). The total sample consisted of 44 interviews with 37 respondents.^2^ Respondents were purposively sampled based on their involvement in HVTs. Potential respondents were approached via email and invited to participate in a face-to-face or telephone interview. Respondents who agreed to participate provided their informed consent for both the interview and its audio recording.

Documents were analysed between July 2009 and August 2012, and interviews were conducted between August 2010 and August 2012.

Interviews were transcribed verbatim and coded on QSR NVivo 10 (a qualitative computer data analysis package). Site documents and interview transcripts were thoroughly coded for HIV prevention practices using a hybrid inductive–deductive approach (Fereday and Muir-Cochrane, 2006) to thematic analysis. The research questions and ethics framework informed an *a priori* coding template. Text was also inductively coded to identify emerging themes. To ensure reliability of coding, a portion of interview data was co-coded by a second researcher. Disagreements were discussed until consensus was reached. A descriptive analysis (devoid of a theoretical framework) (Sandelowski, 2000) was undertaken to describe HIV prevention practices and perceived challenges at sites. Preliminary results were presented at a stakeholder consultation with site staff, CAB and REC representatives.

## Results

This section describes HIV prevention practices for participants at sites as well as reported complexities for each prevention modality. Complexities are clustered according to three major themes in the discussion section. Prevention services provided to individuals not enrolled in trials are also described. Supporting extracts were selected as the most representative of the identified theme.

### Ensuring Access to Risk-Reduction Counselling

Ethics guidelines recommend that trial participants should be provided with appropriate risk-reduction counselling (SAMRC, 2003; UNAIDS-AVAC, 2011; UNAIDS, 2012).

At all sites and for both trials, it was reported that on-site risk-reduction counselling was provided to participants at every visit by trained counsellors. Given that phase I trial participants were at lower risk than phase IIB participants, it was reported that the length and ‘nature of those discussions may be different*’* (Z6, site staff, site 2).

Respondents described counselling sessions as a ‘*personalised*’ risk-assessment where the counsellor can ‘dig more details’ (Z4, site staff, site 2) about a participant’s risk behaviour and jointly develop risk-reduction plans. In addition, detailed risk-behaviour assessments were conducted by members of the research team.

In both trials and at all sites, risk-reduction counselling was guided by a network-provided risk-reduction worksheet. The worksheet comprised an assessment of the participant’s subjective perception of his/her HIV risk as well as an objective exploration of potential risks for HIV/STI acquisition. It also required developing plans for how the participant will reduce his/her risk behaviours and identifying any sources of support including referrals. The worksheet permitted ‘chart-noting’ of counselling sessions, serving as a possible tool for monitoring.

Some sites reported having mentors responsible for training counsellors, debriefing them, providing general support and reviewing ‘*chart-notes*’ of counselling sessions.

#### Reported complexities

*Relying on self-report by participants:* At some sites there was concern that risk assessments relied on self-reports which are notoriously subject to social desirability bias. Since site staff build relationships with participants focused on risk reduction, participants may under-report their risky behaviour to please site staff, for example:

‘So most cases we find people mention that they are using protection. At the end of the day, they not using protection because you find them with STIs, the others are pregnant, others are infected with HIV’ (Z4, site staff, site 2).

Because counselling was tailored to participants’ risk profiles, socially desirable reporting was described as an obstacle to effective risk-reduction counselling. Self-report was also argued to be flawed because participants’ perception of their risk was inaccurate.

*Participants’ implementation of risk-mitigation plans:* However, other respondents observed that participants were forthcoming about their risk behaviours but the difficulty was in implementing risk-mitigation plans:

‘… I'm usually of the view that people are telling us what they’re doing but I guess it is an issue whether or not they are then able to translate whatever insights they reach through the counselling process into some practical steps when they’re outside of the clinic’ (Z6, site staff, site 2).

In terms of correspondence with ethics guidelines, these data suggest that all sites satisfied requirements to provide comprehensive risk-reduction counselling (SAMRC, 2003; UNAIDS-AVAC, 2011; UNAIDS, 2012). Key reported concerns with social desirability are anticipated by select guidelines which outline that site staff should be cognisant of the potential for social desirability bias and recommend the use of neutral advisors and trained counsellors (SAMRC, 2003). Reported complexities also reflect broader concerns with the efficacy of counselling to reduce HIV risk and underscore the search for an expanded array of prevention options that combine biomedical and structural interventions with behavioural interventions (Hankins and de Zalduondo, 2010).

### Ensuring Access to Condoms

Ethics guidelines recommend that participants should receive access to male and female condoms (SAMRC, 2003; UNAIDS-AVAC, 2011; UNAIDS, 2012).

In practice, at all sites and for both trials, male and female condoms were reportedly provided to participants during counselling sessions and participants were counselled to use condoms with every sex act. Condoms were also available outside counselling sessions, for example, at reception or in restrooms. At all sites, condoms were procured from the Department of Health (DoH) at no cost. The risk-reduction worksheet used by all sites allowed ‘chart-noting’ (recording) of condom provision, which was also reported to facilitate feedback to the DoH on the number of condoms issued.

#### Reported complexities

*Ensuring adequate supplies from government partners:* Periodic shortages of government-issued male condoms were reported at two sites:

‘Sometimes you find that the condoms are not available from the provincial office …’ (Z8, site staff, site 4).

‘Well there are times when it’s not available … it would relate to the general shortage of condoms’ (Z10, site staff, site 5).

However, at these sites, staff did not explicitly report that shortages in supply resulted in instances where no condoms could be provided to participants.

At all sites, there were reports about particularly poor accessibility of female condoms, for example:

‘… the majority are really male condoms because to access female condoms is a mission and they are expensive’ (Z22, site staff, site 1).

‘… we didn’t have as many female condoms. I think we had a problem getting the condoms, and we had demands’ (Z11, site staff, site 2).

Given the limited availability, at some sites it was reported that female condoms provision was capped or only provided on request because they ‘… are provided very sparingly from the Department of Health with the proviso that only females who request it are actually dispensed those condoms …’ (C7, site staff, site 4).

*Counsellor promotion of condoms*: Respondents at some sites reported that condom use was emphasized, partly because of the requirement to ‘chart-note’ condom provision on the risk-reduction worksheet:

‘When you counsel someone you have to issue condom, and you have to … chart-note that I issue so much condom to the participant’ (Z2, site staff, site 1).

Since risk-reduction worksheets were reviewed by mentors, it was reported that participants may be pressured by counsellors to take condoms to the extent that participants ‘cannot get out of the [counselling] room without a condom’ (C1, site staff, site 1). Condom provision was described by a respondent at one site as a tick-boxing activity––‘it became a quantitative issue, not a qualitative thing’ (C1, site staff, site 1).

*Low acceptability and uptake by participants*: Respondents at two sites reported that participants have complained that the DoH ‘is not providing worthy condoms’ (Z3, CAB, site 2). Complaints included that these Choice condoms break, are too small and may cause allergic reactions. To remedy concerns, one site secured condoms from an international donor, while at the other site, some participants reportedly opted to purchase their own condoms.

At most sites, there were reports of poor uptake of female condoms. Respondents described various reasons for poor uptake including that it is ‘not comfortable’ (Z7, site staff, site 1), ‘makes a lot of noise’ (Z15, site staff, site 3) and ‘is too big for them’ (Z4, site staff, site 2). Further, ‘they are not user-friendly, you’ve got to put them on quite earlier on, and so those messages are not attractive’ (Z22, site staff, site 1).

In terms of correspondence with ethics guidelines, access to male and female condoms was provided to participants (SAMRC, 2003; UNAIDS-AVAC, 2011; UNAIDS, 2012) at all sites via collaborative efforts with governmental stakeholders (UNAIDS, 2012). Reported challenges with procuring condoms from government partners suggest that constant engagement with such stakeholders is critical. Further, sites should plan for inadequate supply by government partners, given reports that in 2010/11 the DoH fell short of their distribution targets for both male and female condoms (DoH, 2011). The relatively high cost of female condoms may also impede their promotion and provision to the same extent as their male counterpart.

### Ensuring Access to Treatment for STIs

Ethics guidelines specify that participants should be ensured access to STI treatment (SAMRC, 2003; UNAIDS-AVAC, 2011; UNAIDS, 2012).

Across all sites and for both trials, participants were counselled on how to prevent STIs. It was reported that sites followed South African National Guidelines for STI treatment of syndromic management. At four sites participants received STI treatment on-site, while at one site, participants were referred to public healthcare services. If an STI remained unresolved post-treatment, some sites referred participants to the public healthcare sector for further care.

Respondents reported restrictions prohibiting the use of research funding for care services (detailed below). Therefore various strategies were adopted across sites to enable on-site treatment, including procuring from the DoH, and site-funded treatment. It was reported that the network was able to support certain sites to provide on-site treatment, where the site was unable to devise its own strategy.

For the phase I trial conducted at two sites, STIs were reportedly treated on-site with drugs funded by the affected sites. While establishing partnerships to enable on-site dispensing was not always easy, respondents at three sites described that, in future, they will ensure on-site treatment for participants by procuring drugs from the DoH, for example:

‘We have now signed the Memorandum of Understanding with the Department of Health, and we are going to get supplies as a site so that we can be able to provide that …’ (Z15, site staff, site 3).

#### Reported complexities

*Sponsor restrictions of funding*: A key reported challenge was that trial funds come with restrictions:

‘But [sponsor] money is restricted in that it has to be used for research. It cannot be used for care and that is a very clear distinction.’ (C10, network representative)

While complexities with funding were largely described by network representatives, a few respondents at some sites expressed concern about funding restrictions, for example:

‘… there is a clause from the [sponsor] that they cannot spend their money on drugs … at some level it feels a bit like a cop-out … it just seems to be one of those things that you just can’t raise and discuss, so it gets stuck …’ (C11, site staff, site 5)

*Referring to governmental healthcare services:* While a few respondents argued that the referral system ‘is working very well’ (C9, site staff, site 4), at most sites respondents reported challenges, including confidentiality concerns, lack of financial and human resources (which creates long waiting lists) and value-laden and judgmental attitudes of healthcare providers, for example: ‘… they’ll be scolded to ask them “you’re still not using condoms?”’ (Z15, site staff, site 3). Another remarked:

‘… most of the participants still don’t go to the local clinic just because of the way it’s seen. It’s either a family member working there and they don’t want to go there for treatment because then everybody would know’ (Z17, site staff, site 5).

*Using the syndromic management approach*: Respondents at some sites reported complexities with syndromic management, for example: ‘STIs are really over and under treated in our population’ (Z7, site staff, site 1) and if ‘[t]hey don’t report symptoms, we don’t know’ (Z6, site staff, site 2). It was argued that there is a need to develop better methods to diagnose STIs. However, other respondents contended that syndromic management is a better approach in research studies because ‘it enables you to start treatment for a participant prior to getting a laboratory result’ (C7, site staff, site 4).

In terms of correspondence with ethics guidelines, all sites satisfied requirements that participants receive access to STI treatment (SAMRC, 2003; UNAIDS-AVAC, 2011; UNAIDS, 2012). Guidelines do not prescribe whether diagnostic tests or syndromic management should be used. However, the syndromic approach permits healthcare providers to make a timeous diagnosis without specialized skills and sophisticated laboratory tests (Altini and Coetzee, 2005).

### Ensuring Access to Voluntary Medical Male Circumcision

International ethics guidelines require that voluntary medical male circumcision (VMMC) be provided to participants, where indicated (UNAIDS-AVAC, 2011; UNAIDS, 2012).

Across all sites, respondents reported that participants in the phase IIB trial were informed of the benefits of VMMC and that it was provided to all male participants who requested it. For the same trial, circumcisions were paid for by the network via funds sourced from the product developer. For the early-phase trial, VMMC was not paid for by the sponsor nor were alternative funds secured by the network. However, it was reported in interviews that at both sites conducting the phase I trial, VMMC was made available if requested.

At four of five sites, VMMC was ensured through referral to the private sector; and at one site, it was provided on-site by a trained individual. It was argued that referral to the private sector was a strategic decision in order to avoid the challenges of the public healthcare system:

‘… we initially thought of going through the public system … but it’s a mission … people are put on theatre lists and you know how it gets when somebody doesn’t pitch and they don’t get operated that day it’s a mission. So we went the private route which is much easier’ (Z17, site staff, site 5).

#### Reported complexities

*Ensuring access to VMMC across trials*: Respondents at one site asserted that paying for VMMC in one trial, and not in others, created within-site differences between participants enrolled in different protocols:

‘[The phase IIB trial] also funded circumcision for males and none of our other planned or current vaccine trials actually support that. So we do refer people to the public sector with counselling but we don’t have any influence over how soon that care is accessed …’ (C7, site staff, site 4).

However, it was also argued that referral for VMMC would become increasingly acceptable, given scale-up in the public healthcare system.

*Provider promotion of VMMC:* Respondents at some sites expressed concern that low uptake of VMMC may reflect poor provider promotion of circumcision, for example: ‘… there was even a joke of saying that maybe it’s because the investigator sometimes may not really be for circumcision …’ (Z15, site staff, site 3). It was asserted that sound counselling was key to improving uptake of prevention interventions: ‘… if the participants are counselled adequately then the uptake will be good’ (Z7, site staff, site 1).

*Participant acceptability and uptake of VMMC*: At certain sites, the uptake of VMMC was reportedly good. However, at two sites, respondents reported lower uptake, attributed to preferences for traditional circumcisions in the wider community: ‘… you have to realise that we live in a community where male circumcision is part of a custom’ (Z10, site staff, site 5). Therefore, many participants may already be circumcised or may prefer traditional circumcision: ‘many people feel they don’t want to come do it on site, so they will wait for the right opportunity and go and do the traditional way out in the veld’ (Z15, site staff, site 3). Despite concerns about low acceptability and stigma, respondents reported a high uptake of VMMC in those areas with the lowest baseline circumcision prevalence.

In line with international ethics guidelines^3^ (UNAIDS-AVAC, 2011; UNAIDS, 2012), VMMC was provided to all willing participants. Low uptake reported at some sites (attributed to cultural objections) resonates with complexities theorized to be of some importance by ethics commentators (Lie *et al.*, 2006; HPTN, 2009) including that VMMC presents ‘fundamental cultural issues for implementation’ (Haire *et al.*, 2012, p. 23). Low uptake at sites located in traditionally circumcising communities is also consistent with recent research findings (Mark *et al.*, 2012). However, even the low uptake reported in this study suggests that for some individuals from traditionally circumcising communities, offers of VMMC may still be accepted. Therefore, access systems for such services should be considered regardless of the cultural context.

### Ensuring Access to PEP

While South African guidelines (SAMRC, 2003) require that participants be informed about the benefits of PEP and where it can be accessed, international guidelines (UNAIDS-AVAC, 2011; UNAIDS, 2012) require that participants are actually ensured access to PEP, where indicated.

Across all sites, respondents reported that PEP was provided to participants on-site for all risky sexual exposures. During counselling sessions, participants were informed that they need to report to the site ‘within 72 hours’ (Z7, site staff, site 1) and some sites reported implementing strategies to facilitate prompt reporting. At some sites it was reported that the provision of PEP was guided by a site-level standard operating procedure (SOP).

For the phase IIB trial, PEP packs were available on-site (funded by the sites, and/or procured from the DoH or other sources like PEPFAR or provided through the study budget). In some instances, arrangements were brokered with other sites to facilitate access to PEP. For the phase I trial, the two sites covered the cost of on-site PEP provision. The uptake of PEP was reportedly low: ‘it’s not the most popular thing’ (Z18, site staff, site 5) and ‘I can’t remember the numbers exactly, but I think it was pretty small’ (Z9, network representative).

#### Reported complexities

*Initiating PEP using non-government guidelines*: In the public sector the provision of PEP is limited to sexual assaults and not for other risky sexual exposures, like condom failure. However, for the two trials studied here, PEP was provided on-site for all risky sexual exposures:

‘… in South Africa it’s usually given in the public sector for post-rape, post-needle stick injuries, or whatever, but because we’re funded by X [name of sponsor] … they follow the US sort of guidelines with regard to HIV prevention’ (Z7, site staff, site 1).

Some respondents questioned the appropriateness of providing PEP to participants for all sexual exposures, given that it is not in line with national policy.

A respondent at one site stated that offering PEP for all risky sexual exposures in some trials but not in others created differences between participants enrolled in different protocols at the same site.

‘I think the one challenge I’ve experienced and this had been a difference between [the phase IIB trial] and our other vaccine trials is that in [the phase IIB trial] we provided post exposure prophylaxis for risky sexual contact, it didn’t have to be a sexual assault but for all our other protocols we follow the national department of health guidelines because PEP is not provided by the protocol budget.’ (C7, site staff, site 4).

Furthermore, at another site it was reported that where PEP was not ensured through the protocol budget, the site would self-fund PEP for all risky sexual exposures. It was acknowledged that site-funded PEP provision may not always be possible (e.g., for less-resourced sites), raising the potential issue that if trials and/or sites adopt different strategies (e.g., referral to public healthcare services versus on-site provision), different standards might be introduced for participants within or across sites (e.g., only initiating PEP for sexual assault versus for all risky sexual exposures).

*Provider promotion of PEP*: Some respondents expressed concern about the lack of evidence from randomized controlled trials to support the efficacy of PEP as well as safety concerns, for example: ‘… we don’t have direct evidence that it works’ (Z6, site staff, site 2) and ‘[PEP provision is] not necessarily in the patient’s greatest safety interests’ (Z16, REC member). However, others suggested that there was sufficient evidence to support the use of PEP.

A respondent at one site reported that PEP was provided inconsistently because of provider beliefs about efficacy: ‘some people provide it, some people don’t’ (Z6, site staff, site 2) and described PEP as ‘a very un-standard part of the study’ (Z6, site staff, site 2). Respondents at two sites reported that the use of SOPs may improve standardization. Further, at two sites, it was described that counselling about PEP was not as intensive as for other preventive interventions.

The provision of PEP for all sexual exposures exceeded South African guideline recommendations but complied with international guidelines. While such services may not be sustainable post-trial in this setting, it has been argued that the immediate potential benefit of reduced HIV infection risk for participants is a legitimate trial-related benefit (Haire *et al.*, 2013).

### Ensuring Prevention Services for Non-Enrolled Persons

Guidelines do not comprehensively describe the standard of prevention owed to persons not enrolled in trials, except the provision of couples counselling (UNAIDS-AVAC, 2011) and informing participants about how to obtain STI treatment for their partners (SAMRC, 2003). In addition, UNAIDS-AVAC (2011) recommends consultation on the specific HIV prevention services that will be available to participants’ partners. There are also general statements that screen-outs should be referred, where relevant (SAMRC, 2003) and provided with information about HIV prevention services available in the community (UNAIDS, 2012).

All sites reported providing some prevention services to volunteers at screening, partners of participants and the wider community. Both protocols outlined that risk-reduction counselling was provided at screening. In addition, some sites reported providing counselling to partners via couples counselling, to community members at the level of voluntary counselling and testing and broader HIV prevention education. Condoms were freely available at sites and also dispensed at community events.

It was reported that volunteers presenting for the phase I trial were screened for syphilis and if infected, were not enrolled. Some sites reported that for the phase IIB trial, volunteers with STIs at screening were enrolled after successful treatment. Various mechanisms for ensuring access to STI treatment at screening were adopted across sites and for both trials, including on-site provision (where funds permitted), or referral to a co-located or public healthcare facility. A few respondents reported that participants were informed about where their partners could access treatment. However, most respondents across all sites reported that partners were referred to the public sector, with a ‘yellow card’ or referral letter/slip.

For the phase IIB study, a circumcision assessment was conducted at screening and volunteers were informed of the benefits of circumcision. PEP was reportedly only ensured for enrolled participants. Respondents at some sites described that the provision of prevention services to non-enrolled persons is difficult in the context of constrained trial budgets.

Some REC respondents argued that the researcher’s ‘principal obligation is in fact to the participant’ (Z16, REC) and that there are more limited obligations to non-trial participants, including HIV prevention education and referral to appropriate services.

Sites have generally exceeded guideline requirements by ensuring access to certain prevention services for non-trial participants. Previous research has found that trial sites endeavour to provide a variety of services to partners and screen-outs but noted that this may make conducting research prohibitively expensive (Ngongo *et al.*, 2012). Similarly in this study, resource and budget constraints were reported as impeding the provision of prevention interventions to non-trial participants.

Guidance is unclear on the standards of prevention for those not enrolled in trials (Tarantola *et al.*, 2007). Therefore, it has been recommended that researchers and sponsors should make such determinations in consultation with relevant stakeholders (Tarantola *et al.*, 2007; Ngongo *et al.*, 2012).

### Stating Plans to Ensure Access to Services

Ethics guidelines (SAMRC, 2003; UNAIDS, 2012) require that risk-minimization measures be outlined in the protocol, and the informed consent form and process.

The phase IIB protocol stated that the coordinating network ‘*is committed to ensuring that all trial participants receive access to the highest standard of prevention*’ including risk-reduction counselling, male and female condoms, syndromic management of STDs, information on and referral to VMMC services and PEP when indicated. The phase I protocol only specified that risk-reduction counselling would be provided to participants.

Informed consent forms for both studies specified that counselling would be provided to participants but provided no information about other prevention options. It was reported that this may either be an oversight or deliberate to allow for flexibility in REC review, for example: ‘the other thing we don’t want to do is be too specific/so, every time there’s a change to the consent form, obviously it has to go back to the ethics committee for review’ (Z9, network representative). However, respondents reported that HIV prevention components are described to participants in the informed consent process.

## Discussion

This research aimed to explore the extent to which actual practices in HVTs corresponded with related recommendations in ethics guidelines, to identify complexities with implementation of services and to make possible recommendations for guideline revisions and strengthened practices.

Ethics guidelines required that participants be provided with access to a comprehensive package of HIV prevention interventions, described as ‘state-of-the-art’ (UNAIDS-AVAC, 2011; UNAIDS, 2012) or ‘optimal’ (SAMRC, 2003). The HVTs in the present study satisfied these ethics recommendations by providing risk-reduction counselling and access to male and female condoms and STI treatment (SAMRC, 2003; UNAIDS-AVAC, 2011; UNAIDS, 2012) as well as VMMC and PEP, where indicated (UNAIDS-AVAC, 2011; UNAIDS, 2012). In this way, these trials intertwined ‘biomedical research with public health practice’ (Macklin, 2010, p. 203). In some instances, practices at sites exceeded guideline recommendations, for example, by paying for VMMC at private facilities. Site services also sometimes exceeded preventive options available in the local community, for example, by ensuring access to PEP for all sexual exposures, and to VMMC at a time of limited public access. This indicates that, in the setting in which these data were collected, ethics recommendations to provide ‘state-of-the-art’ prevention services were in fact achievable.

Ethics guidelines recommend that the provision of risk-reduction interventions be monitored (SAMRC, 2003; UNAIDS, 2012). Many reported practices could reflect monitoring activities, such as recording condoms dispensed, recording STIs and their resolution and recording uptake of PEP and VMMC—all collated in participants’ records. This study found that practices deviated from guidelines in terms of the content of consent forms and protocols—sites provided substantially more to participants than was specified in the consent documents for both trials and in the phase I trial protocol.

Reported complexities identified with the implementation of each prevention modality can be clustered according to three major themes, namely, (i) partnerships/funding; (ii) provider promotion; and (iii) participant acceptability and uptake. In terms of partnerships/funding, these findings suggest that sponsor restrictions have implications for the provision of prevention services. More specifically, for STI treatment, sites had to engage service providers to dispense STI treatment on-site, or themselves raise funds; alternatively they made referrals to the public sector. Further, the network raised funds to pay for VMMC in one trial, and the sites ensured access in the other trial. Restrictions on sponsor funds for care provision not necessary for scientific validity or participant safety (Philpott *et al.*, 2010) have led researchers to partner with other stakeholders in ensuring access to key prevention services. In line with UNAIDS (2012) guidelines, these data indicate that the burden of providing prevention interventions is shared among sponsors, researchers, government service-providers and, in some instances, private donors.

This study found that access to VMMC was ensured for both trials and at all sites (on-site or via referrals to private facilities), and identified the reported concern that other concurrent or overlapping trials may adopt different approaches to delivering VMMC (e.g., referral to public health facilities), potentially raising the issue of fairness in adopting different strategies across trials.

This study found that access to PEP was ensured for all risky sexual exposures for the two trials at all sites, and identified the reported concern that for some trials and some sites, this approach may not be possible (for example, where sites do not have the resources to self-fund PEP). This raises the concern that where trials and sites use different approaches for PEP, this may create differences between participants in different protocols at the same site or between participants enrolled at different sites.

Ethics guidelines assert that protocols may vary in ‘modes of delivery’ for prevention interventions (UNAIDS, 2012, p. 45), that negotiations should occur on a trial-by-trial basis (SAMRC, 2003; UNAIDS-AVAC, 2011; UNAIDS, 2012) and that when funding restrictions limit which interventions can be provided, researchers should find alternative funding or partner to ensure access (UNAIDS-AVAC, 2011). This suggests that guidelines allow for some flexibility in approaches to the implementation of standards of prevention.

Findings from this study indicate that referral is not a common strategy for the delivery of prevention services, with most prevention services provided on-site. However, the DoH emerged as a key partner in ensuring access to many prevention services. Engaging this partner was associated with some tensions, for example, late delivery of condoms from government, or constraints bearing on STI treatment at public sector facilities, as predicted by literature (Chatterjee *et al.*, 2006).

In terms of provider promotion, this study identified concerns about mechanical promotion of condoms, anxieties about promoting VMMC in certain cultural contexts and concerns about the efficacy of PEP—all of which point to the critical role of provider attitudes on uptake of services (Bharat and Mahendra, 2007; Hoffman *et al.*, 2004). Participant’s autonomous preferences should be enabled—by skilled and motivated providers—even when such decisions are based on values or world views that do not accord with those of the provider (Entwistle *et al.*, 2010).

In terms of participant uptake, this study identified low uptake of female condoms, and of VMMC in some cultural contexts, which suggest that in some instances, despite availability and promotion, some participants may elect not to take up prevention options that are not in line with their values and preferences. This underscores the need for a ‘menu of options’ that can be tailored to the needs of individuals and sub-groups (Chatterjee *et al.*, 2006).

## Limitations

Direct observations of HIV prevention services at sites were not conducted. Trial participants were not sampled in this study, even though they were likely to provide an additional perspective on the questions addressed in this article. However, this study’s sample and methodology were designed to facilitate critical reflection about prevention services rather than to audit ‘end-users’. It was hoped that CAB members, as a proxy for the community, would provide some indication of the experiences of participants. This study only sampled South African sites and was limited to two trials. However, it is hoped that the issues identified here might be usefully applied to similar trials in other settings.

## Conclusions

Despite concerns that ethics guidelines set the bar high (HPTN, 2009; Macklin, 2009) and may be infeasible especially in resource-constrained contexts, this study found a high degree of correspondence between actual practices at South African HVT sites and related recommendations in ethics guidelines. However, practices deviated from guidelines regarding the drafting of consent materials. Data from this study also indicate that while ethics guidelines anticipate some of the core thematic complexities raised (namely, funding), challenges regarding provider promotion and participant uptake of prevention interventions are not adequately addressed by guidelines.

## Recommendations

This study aimed to make recommendations for improved practices at sites. Complexities reported with the implementation of prevention interventions may be of relevance as new prevention methods, e.g., PrEP, become accepted as part of the standard of prevention. It is recommended that stakeholders carefully consider the partnerships that need to be established to enable access to upcoming prevention tools, for example, to procure products for on-site dispensing or to establish referral relationships. Given findings that site staff promotion of prevention interventions may be impacted by the cultural context and perceptions of the efficacy of the intervention, sites should consider implementing formal mechanisms to assess provider promotion of prevention services and to train staff to ensure consistent promotion. Strategies should be developed to recognize and respect objections to uptake from certain sub-groups based on values and preferences.

These data serve to supplement already-flagged concerns regarding upcoming technologies, such as establishing scientific or clinical validity (Jay *et al.*, unpublished manuscript), appropriate regulatory approval, ensuring adequate community consultation and considering interaction effects (Dawson, 2012; Haire *et al.*, 2012; Macklin, 2012). Data about tensions related to engaging key partners suggest that new sites should engage critical stakeholders such as the DoH in an early, sustained and strategic manner. It also suggests the usefulness of ongoing evaluation of the quality of key partnerships. The data reflecting challenges in funding certain prevention interventions suggest that sponsor restrictions on how funding can be utilized need to be revisited (Philpott *et al.*, 2010).

Data indicated that consent forms contained fewer disclosures about prevention options than were actually provided to participants. While the form is only part of the informed consent process (Flory and Emanuel, 2004), given that participants may use the consent form for reference purposes (Ramjee *et al.*, 2010), it is recommended that they contain more information on the standard of prevention. This may also help counter potential variability in provider promotion of services. More detailed disclosures in consent forms should, where possible, preserve site flexibility in implementing services.

This study also aimed to make recommendations for ethics guidance. Data about perceived complexities regarding provider promotion and participant uptake suggest that guideline recommendations to ‘monitor’ prevention interventions should be refined to recommend evaluation of both ‘promotion’ and ‘uptake’. Future revisions should make recommendations that address challenges identified empirically, such as provider promotion. It is also recommended that South African ethics guidelines be updated to reflect recent evolutions in HIV prevention.

While recommendations to aspire to ‘state-of-the-art’ prevention services were intended to minimize so-called double standards between developed and developing countries (Haire *et al.*, 2013), data indicate the potential for different standards between participants enrolled in different protocols and/or at different sites. This suggests that ethics guidelines will need to grapple with the issue of different standards between protocols implemented within the same site or between sites within the same country. Given that efforts to reduce the risk of HIV acquisition among trial participants is of great concern to community stakeholders (UNAIDS-AVAC, 2011), constant monitoring of the outcomes of various approaches should be undertaken and communicated to key stakeholders.

While guidelines are generally silent on whether the obligation to provide prevention services differs according to the phase of the trial or risk level of participants, these data indicate that protocol writers accord different obligations to early-phase and late-phase trial participants. Future revisions should consider clarifying whether obligations to participants differ based on trial phases.

These data also identified that trial sites endeavour to provide prevention interventions to persons not enrolled in trials. However, ethical guidelines provide little direction on this issue. Future revisions should be clearer on what, if anything, should be ensured for those not enrolled in trials.

## Notes

1. The UNAIDS *Ethical Considerations in Biomedical HIV Prevention Trials* guidance document was originally published in 2007. An additional guidance point was added in 2012.

2. The terms interviewee and respondent indicate those who participated in interviews for the present study, while the term participant is used for individuals who participate in HVTs. Seven respondents participated in both the prevention-specific and the care-specific interviews.

3. VMMC is not mentioned in South African guidelines because they were published in 2003 before male circumcision was proven as an effective HIV risk-reduction intervention.
